# Risk Factors of Birth Asphyxia among Newborns at Debre Markos Comprehensive Specialized Referral Hospital, Northwest Ethiopia: Unmatched Case-Control Study

**DOI:** 10.4314/ejhs.v32i3.6

**Published:** 2022-05

**Authors:** Yoseph Merkeb Alamneh, Ayenew Negesse, Yared Asmare Aynalem, Wondimeneh Shibabaw Shiferaw, Mihretie Gedefew, Melkamu Tilahun, Yidersal Hune, Abtie Abebaw, Yalemgeta Biazin, Tadesse Yirga Akalu

**Affiliations:** 1 School of Medicine, Debre Markos University, Debre Markos, Ethiopia; 2 College of Health Science, Debre Markos University, Debre Markos, Ethiopia; 3 College of Health Science, Debre Berihan University, Debre Berihan, Ethiopia

**Keywords:** Birth asphyxia, Case-control study, risk factors, Ethiopia, Newborn

## Abstract

**Background:**

Despite a global decline in under-five deaths, the neonatal mortality rate remains slow in developing countries and birth asphyxia remains the third cause of neonatal deaths. Globally, neonatal deaths accounts for 45% of under-five deaths, birth asphyxia causes 23–40% of neonatal deaths in Ethiopia. There is limited data on risk factors of asphyxia in Ethiopia, particularly in the study area. Therefore, this study aimed to identify the risk factors of birth asphyxia among newborns.

**Methods:**

This research followed a hospital-based unmatched case-control study design at Debre Markos comprehensive specialized referral hospital, Northwest Ethiopia, among 372 newborns (124 cases and 248 controls). Data were collected by interviewing index mothers and chart review using a pre-tested questionnaire. Then it was entered in Epi-data version 3.1 and transferred to STATA version 14.0 for analysis. Bivariate and multiple variable logistic regression were carried out to the possible risk factors. Finally, statistical significance was declared using adjusted odds ratio with 95% CI and p-value <0.05.

**Results:**

Prolonged labor >12, meconium-stained amniotic fluid, assisted vaginal delivery, gestational age < 37 weeks, noncephalic presentation, comorbidity, birthweight<2500grams were found to be significant factors of birth asphyxia.

**Conclusion:**

In this study, Prolonged labor >12 hours, meconium-stained amniotic fluid, assisted vaginal delivery, gestational age < 37 weeks, non-cephalic presentation comorbidity, fetal distress, birthweight<2500grams were found to be risk factors of birth asphyxia were risk factors of birth asphyxia. Therefore, to reduce neonatal mortality associated with birth asphyxia, attention should be given to holistic pregnancy, labor and delivery care, and post-natal care. Moreover, interventions aimed at reducing birth asphyxia should target the identified factors.

## Introduction

Birth asphyxia is the difficulty to initiate and maintain breathing at birth which is distinguished by a marked impairment of gas exchange; if sustained leading to progressive hypoxemia and substantial metabolic acidosis (1,2). Globally, around 4 million under-five children die per year of which 45% occurs during the neonatal period, and 75% of neonatal deaths occurs in low-income countries (3–5). In developing countries, neonatal deaths accounted for 52% of all under-5 mortality. However it is due to preventable causes including perinatal asphyxia (6,7). In Ethiopia, birth asphyxia is one of the driving causes of neonatal mortality, constituting 34% (7). Besides, Findings from Nigeria (8), Southern Nepal (9), and Khulna Urban Slum, Bangladesh (10) indicate that birth asphyxia is blamable for about 23.9%, 30%, and 39% of neonatal deaths, respectively. Neonatal consequences following birth asphyxia include multisystem organ dysfunction, neonatal neurological problems such as seizure, coma, and hypotonic (neonatal encephalopathy) (4,11). The complication of birth asphyxia is not restricted only to death but also leads to physical, mental and social inability in neonates as a result of severe hypoxic-ischemic encephalopathy (12). Birth asphyxia is caused by multifaceted factors, categorized as antepartum factors (50–70%), intrapartum factors (20–40%) and post-partum factors (10%) (13,14). Common risk factors of birth asphyxia include maternal age under 16 or over 35 years old, gestational age <37 or >41 weeks, diabetes, drugs and alcohol, hypertensive disorders, bleeding in the second or third trimester, prolonged labor, cesarean section, instrumental delivery, prolonged and premature rupture of membranes, meconium staining, maternal infection and comorbidity during pregnancy (15,16).

Commonly known determinants of birth asphyxia vary across regions, depending on the context. In Ethiopia, where birth asphyxia is the second leading cause of neonatal mortality, early recognition and management of the risk factors of birth asphyxia are pillars to reduce neonatal deaths and improve neonatal quality of life. Therefore, it is necessary to identify the specific risk factors in a particular region to take appropriate interventions. Thus, this study aimed to identify the risk factors of birth asphyxia among newborns at Debre Markos referral hospital, Northwest Ethiopia. The findings of this study will be used as input for policymakers and program planners who work in the area to inform, plan, implement and evaluate health promotion policies and strategies on the reduction of under-five children mortality and improvement of child health care.

## Subjects and Methods

**Study design, period and setting**: Institutional-based unmatched case-control study design was conducted considering neonates with asphyxia as cases and those without asphyxia as controls among newborns from August 1/2019 to October 2019 at Debre Markos referral hospital which is located in Debre Markos town, East Gojjam Zone, Amhara regional state. The town is found 299 km north west from Addis Ababa, capital city of Ethiopia. According to information obtained from the administrative offices of Debre Markos comprehensive specialized referral hospital, they provide different services in the outpatient department, inpatient department and operation room theatre department. The hospital serves for more than 3.5 million populations in the catchment area and it has more than 30 beds in NICU with annual admission of more than 2800 neonates of which, more than 770 neonates are by birth asphyxia. There are five pediatricians and 21 nurses in NICU. In obstetrics and gynecology ward there 7 gynecologists and 45 midwifery professionals with the annual delivery 6734 neonates.

**Study participants**: The study population was both neonates with asphyxia and those without asphyxia admitted to neonatal intensive care units (NICU) in Debre Markos referral hospital. All live newborns who were born after 28 weeks of gestation were screened for eligibility. The study classified participants into cases and controls. Newborn babies with < 7 APGAR scores at 5 minutes were defined as having asphyxia at birth (cases), while newborn babies with ≥7 APGAR scores at 5 minutes were considered non-asphyxiated (controls). The research included newborns who met the case-control definition and whose mothers gave informed consent. The study excluded newborns who had met the case- control definition but whose mothers had not been interviewed voluntarily. Newborns with one or more life-threatening malformations, including congenital cyanotic heart defects and hydrops, and other severe birth defects also excluded. Besides, newborns with no recorded of 5^th^ minute APGAR score and who were not diagnosed as birth asphyxia were excluded from the study.

**Sample size calculation and sampling techniques**: The sample size was estimated using an unmatched case-control formula (Kelsey), which assumed a confidence level of 95%, power (80%) and case-control ratio of 2, and odds ratio (OR) of 2 to be defined as significant using Epi-info7. The proportion of non-asphyxia neonates (controls) (31.3%) and asphyxia cases (60.0%) with prolonged labor exposure (>12 hours) were obtained from a previously conducted study (17) and the total sample size was obtained by adding 10% nonresponse rate, which was 372 (124 cases and 248 controls). Cases and controls were recruited on a continuous basis between August 1, 2019 and October 30, 2019 until the appropriate sample size was reached for both groups.

**Data collection tool and procedure**: Data were collected using a pre-tested and adapted standardized questionnaire (18) it was administered by interviewers, observational and chart review was used to gather data on sociodemographic maternal variables, variables related to obstetric history (mother's age, education, pregnancy number, parity, history of pregnancy outcome, gestational age; antepartum factors (prime parity, maternal fever, pregnancy-induced hypertension, anemia, peripartum hemorrhage, history of previous neonatal deaths); intrapartum factors (mal-presentation, prolonged labor, meconium-stained liquor, pre-eclampsia, eclampsia, augmentation of labor, complicated labor, mode of delivery); fetal factors (sex, birth weight, the maturity of the newborn). The tools were prepared in English and translated to Amharic; eventually, it was translated back to English to check the consistency. Birth asphyxia was determined using APGAR score which consisted of five components such as appearance (color), pulse (heart rate), grimace (reflexes), activity (muscle tone) and respiration, each with a score of 0, 1, or 2. A score of (≥7) showed no asphyxiation of a newborn while a low score (< 7) revealed an asphyxiated newborn (17).

**Data quality control**: The quality of the data was ensured by using properly designed data collection tools. Training was given for data collectors and supervisors for two-days on data collection procedures, techniques and methods. Prior to data collection, the questionnaire was tested in five percent (7 cases and 14 controls) at Lumame hospital to verify the questioner's accuracy. Clarification of question and time to complete the questionnaire was assessed. The supervisors and the principal investigator reviewed and updated the computed questionnaires every day, and the data collectors provided the necessary input for the next morning before the actual procedures began.

**Study variables**: Birth asphyxia was the dependent variable. While the independent variables include maternal characteristics and variables related to obstetric history (mother's age, education, pregnancy number, parity, history of pregnancy outcome (singleton or multiple ), gestational age; antepartum factors (prime parity, maternal fever, pregnancy-induced hypertension, anemia, peripartum hemorrhage, history of previous neonatal deaths); intrapartum factors (mal-presentation, prolonged labor, meconium-stained liquor, pre-eclampsia, eclampsia, augmentation of labor, complicated labor (cord prolapse), mode or type of delivery); fetal factors (sex, birth weight, the gestational age of the newborn).

**Data analysis**: Data was entered in Epi-data version 3.1 and transferred to STATA version 14.0 for analysis. Using descriptive statistics, socio-demographic factors, antepartum, intrapartum, and neonatal- related factors are presented using frequency tables, figures, and percentages. In the second stage, bivariate logistic regression was used to identify possible factors of candidate variables with a p-value <0.2 for the final model. The model fitness test was carried out using the Hosmer – Lemeshow test, which is a statistical test for fitness for logistic regression models. Finally, the multivariable logistic regression model was fitted to identify significant risk factors of birth asphyxia through a backward stepwise method, risk factors of birth asphyxia among newborns were determined using their adjusted odds ratio with 95% CI and p-value < 0.05.

**The following operational definitions are used Birth asphyxia**: Neonate failure to start and sustain sufficient respiration within 5 minutes of birth with an Apgar score below 7 (19).

**Cases (asphyxiated newborns)**: all neonates diagnosed with asphyxia by the attending health professionals using an Apgar score of less than 7 at 5 minutes after birth were considered as cases. Controls (non-asphyxiated newborns) – all neonates diagnosed as non-asphyxiated by the attending health professionals using an APGAR score of more than 7 at 5 minutes were considered as controls.

**Ethics Approval and Consent to Participate:** The ethical clearance letter has been received from the research and review committee from college of health sciences, Debre Marks University. Additionally, prior to beginning data collection permission was obtained from the hospital authority. Finally, informed written consent was received from each participant mothers after explaining the research objectives. The participants were briefed on the study's purpose, procedures, potential risks, and benefits. In addition, the participants were told that failure to agree or to withdraw from the study would not change or endanger their access to treatment.

## Results

**Socio-demographic characteristics**: In this study, a total of 372 newborns (124 cases and 248 controls) were included in the analysis. Regarding the age of index mothers, majority (71.8 %) of cases and nearly half (49.6%) of controls were in the age group of 19 – 34 years. By residence of mothers, more than half (67.7%) of cases were from rural area. But, majority (75.5%) of controls were from urban residence. Most of the study participants were Orthodox Christian followers (88.7% cases and 89.9% controls). By marital status, majority of index mothers (84.7% cases and 78.6% controls) were married. Twenty-three (23.4%) of the cases mothers and 24.2% of the controls did not have formal education (Table 1).

**Table 1:** Distribution of socio-demographic characteristics of cases and controls attending in Debre Markos referral hospital, Northwest Ethiopia 2019

Variables	Response Category	Birth asphyxia	

		Yes (cases)	No (controls)
Residency	Urban	40 (32.3)	187 (75.4)
	Rural	84 (67.7)	61 (24.6)
Age	≤ 18	20 (16.1)	43 (17.3)
	19–34	89 (71.8)	123 (49.6)
	≥ 35	15 (12.1)	82 (33.1)
Marital status	Married	105 (84.7)	195 (78.6)
	Unmarried	19 (15.3)	53 (21.4)
Religion	Orthodox	110 (88.7)	223 (89.9)
	Muslim	12 (9.7)	22 (8.9)
	Protestant	2 (1.6)	3 (1.2)
Ethnicity	Amhara	120 (96.8)	242 (97.6)
	Oromo	4 (3.2)	6 (2.4)
Occupation	Government	25 (20.2)	74 (29.8)
	Private	20 (16.1)	55 (22.2)
	Merchant	15 (12.1)	33 (13.3)
	Housewives	52 (41.9)	44 (17.70
	Student	12 (9.7)	42 (16.9)
Education	Primary	50 (40.3)	71 (28.6)
	Secondary	21 (16.9)	64 (25.8)
	College	24 (19.4)	53 (21.4)
	Not read and write	29 (23.4)	60 (24.2)

**Maternal and antepartum related factors**: Sixty-nine (55.60 %) of the mothers in the cases and (48.8 %) of the controls were multipara. There was a higher rate of > 4 antenatal care (ANC) visits among cases (52.4 %) than controls (41.1%), and the incidence of comorbidity among cases during pregnancy (38.7 %) was higher in proportion than controls (24.2 %). A duration of pregnancy < 37 weeks of gestation was more common among cases (32.3 %) than controls (11.2 %) (Table 2).

**Table 2:** Characterization of cases and controls of antepartum and intrapartum and fetal factors of birth asphyxia among newborns delivered in Debre Markos referral hospital, Northwest Ethiopia 2019

Variables	Response Category	Birth asphyxia	

		Yes (cases)	No (controls)

		Count (%)	Count (%)
Gravidity	Primi	55 (44.4)	127 (51.2)
	Multi	69 (55.60)	121 (48.8)
ANC follow up	Yes	65 (52.4)	102 (41.1)
	No	59 (47.6)	146 (58.9)
Comorbidity during pregnancy	Yes	48 (38.7)	60 (24.2)
	No	76 (61.3)	188 (75.8)
Fetal presentation	Noncephalic	36 (29.0)	36 (14.5)
	Cephalic	88 (71.0)	212 (85.5)
Mode of delivery	Assisted/instrumental	42 (33.9)	33 (13.3)
	SVD	82 (66.1)	215(86.7)
Prolonged labor	> 12hr	86 (69.4)	83 (33.5)
	≤ 12hr	38 (30.6)	165 (66.5)
Labor type	Induced	24 (19.4)	38 (66.5)
	No induced	100 (80.6)	210 (84.7)
Meconium-stained amniotic fluid	Yes	46 (37.1)	37 (14.9)
	No	78 (62.9)	211 (85.1)
Gestational age	< 37weeks	40 (32.3)	31 (12.5)
	≥ 37weeks	84 (67.7)	217 (87.5)
Fetal Outcome	Multiple	30 (24.2)	43 (17.3)
	Single	94 (75.8)	205 (82.7)
Fetal distress	Yes	53 (42.7)	48 (19.4)
	No	71 (57.3)	200 (80.6)
Birth weight	< 2500	69 (55.6)	90 (36.3)

**Intrapartum related characteristics and neonatal/ fetal related factors**: The non-cephalic fetal presentation was relatively common among cases (29.0 %) compared with controls (14.5 %). There was also a disparity in proportion in terms of prolonged labor (>12 hours), where (69.4%) of cases were born after prolonged labor and only (33.5%) of controls were born after prolonged labor. Forty-six (37.1 %) cases and (14.9%) controls had meconium-stained liquor on pelvic examination. Forty (32.3%) of the cases and only (12.5 %) of the controls were preterm; (55.6 %) and (36.3 %) were low birth weight. There were (24.2 %) twin newborns among the cases and (17.3 %) among the controls (Table 2).

**Risk factors of birth asphyxia**: Bivariate analysis was performed in binary logistic regression analysis to analyze the possible risk factors of birth asphyxia, and variables with a p-value of ≤ 0.2 were entered in the multivariable model. The analysis of bivariate logistic regression showed that educational status, gravidity, ANC follow-up, co-morbidity during pregnancy, fetal presentation, instrumental delivery, prolonged labor, type of labor, meconium-stained amniotic fluid, gestational age, fetal outcome, fetal distress, and birth weight were associated with birth asphyxia.

Multivariable logistic regression was performed by simultaneously taking into account those variables. Stepwise the backward regression elimination process was used to assess the confounding variables. Finally, in multivariable logistic regression, co-morbidity during pregnancy, non-cephalic fetal presentation, instrumental delivery, prolonged labor, meconium-stained amniotic fluid, gestational age, fetal distress and birth weight at p<0.05 at 95% CI after adjustment for potential effects of confounding variables were found to be associated with birth asphyxia.

It was found that meconium-stained amniotic fluid has a significant association with the risk of birth asphyxia. In particular, neonates delivered from mothers with meconium-stained amniotic fluid were almost four times more likely than counterparts to develop birth asphyxia (AOR = 4.25, 95 % Cl; 2.67, 6.73). The likelihood of developing birth asphyxia among neonates born from prolonged-labor mothers (> 12 hours) was around three times higher than their counterparts (AOR = 2.69, 95 % CI: 1.88, 4.63). Neonates with intrapartum fetal distress were nearly five times more likely to experience asphyxia at birth than those born with normal fetal heart rate (AOR = 5.45; 95 % Cl: 2.48, 7.19). Instrumental delivery (vacuum or forceps) was found to be significantly associate with the occurrence of birth asphyxia. Specifically, neonates born with the help of vacuum or forceps were around three times more likely to be asphyxiated compared to neonates born through spontaneous vaginal delivery (AOR=2.80, 95% CI:1.20, 3.39). Neonates born with low birth weight were around four times more likely to have birth asphyxia compared with those born with normal weight (AOR = 4.20, 95 % CI: 2.78, 6.71). Concerning fetal presentations, newborns with the non-cephalic presentation were nearly five times more likely than those with a cephalic presentation to experience birth asphyxia (AOR: 5.20, 95% CI: 3.27, 8.28). Also, neonates born from mothers with co-morbidity during pregnancy were around three times more likely than their counterpart to develop birth asphyxia (AOR=3.40, 95% CI: 1.52, 3.67). Furthermore, preterm babies were around three times more likely to develop birth asphyxia than the term (AOR = 2.60, 95 % CI:1.66, 4.45) (Table 3).

**Table 3:** Bivariate and multivariable logistic regression among factors of cases and controls attending Debre Markos referral hospital, Northwest Ethiopia 2019

Variables	Response category	Birth Asphyxia		COR (95% CI)	AOR (95% CI)

		Yes (cases)	No (Controls)		
Residency	Urban	69	187	1	1
	Rural	55	61	2.44(1.55, 3.86)	-
Education	Not read and write	29	60	2.15(1.164, 3.96)	-
	Primary	50	71	1.56 (.85, 2.84)	-
	Secondary	21	64	1.46(.822,2.58)	-
	College	24	53	1	1
Gravidity	Primi	55	127	1.32(.85, 2.03)	-
	Multi	69	121	1	1
ANC follow up	Yes	65	102	1	1
	No	59	146	1.56 (1.022, 2.433)	-
Comorbidity during pregnancy	Yes	48	60	1.98 (1.25, 3.15)	3.40(1.52, 3.67)
	No	76	188	1	1
Fetal presentation	Noncephalic	36	36	2.41 (1.43, 4.07)	5.20 (3.27, 8.28)
	Cephalic	88	212	1	1
mode of delivery	Assisted/instrumental	42	33	3.34(1.98, 5.63)	2.80(1.20, 3.39)
	Svd	82	215	1	1
Prolonged labor	> 12hr	86	83	4.45 (2.83, 7.16)	2.69(1.88, 4.63)
	≤ 12hr	38	165	1	1
Labor type	Induced	24	38	1.33 (.76, 2.33)	-
	None	100	210	1	1
meconium-stained amniotic fluid	Yes	46	37	3.37(2.03, 5.57)	4.25(2.67, 6.73)
	No	78	211	1	1
Gestational Age	< 37wk	40	31	3.33 (1.96, 5.68)	2.60 (1.66, 4.45)
	≥ 37wk	84	217	1`	1
Outcome	Multiple	30	43	1.52 (.90, 2.58)	-
	Single	94	205	1	1
Fetal distress	Yes	53	48	3.11 (1.93, 5.00)	5.45(2.48, 7.19)
	No	71	200	1	1
Birth weight	< 2500	69	90	2.20 (1.42, 3.42)	4.20 (2.78, 6.71)
	≥ 2500	55	158	1	1

## Discussion

Birth asphyxia is one of the leading cause of mortality for newborns; the effect of birth asphyxia is not limited only to death but also leads to physical, mental and social incapability in newborns due to severe hypoxic-ischemic organ damage (12). Hence, to reduce the overall newborn mortality and its long-term consequences the quality of medical care before birth, at birth, and after birth is essential. This research is important to understand the determinant factors of birth asphyxia. It has been attempting to look at the risk factors of birth asphyxia by incorporating as many factors as possible. Birth asphyxia was found to be significantly associated with co-morbidity during pregnancy, non-cephalic fetal presentation, instrumental delivery, prolonged labor, meconium-stained amniotic fluid, gestational age, fetal distress and low birth weight.

The odds of birth asphyxia were 4.25 times higher among neonates born from mothers with a history of meconium-stained amniotic fluid. This finding was similar to previous studies in Uganda, India, and Sweden (20–22). The plausible reason could be that meconium-stained amniotic fluid occurs in peripartum inhalation with meconium-stained amniotic fluid, contributing to chemical pneumonia with pulmonary tissue swelling, mechanical airway congestion, and pulmonary air loss, leading to hypoxia (23). The likelihood of developing birth asphyxia among neonates born from mothers with prolonged-labor (> 12 hours) was 2.69 times higher than their counterparts. This result is compatible with the results of a study conducted in Dire Dawa and Malawi (24, 25). This may be since, labor does not proceed normally a mother may suffer serious complications, such as maternal and neonatal infection dehydration exhaustion or rupture of the uterus and fetus, which may contribute to the birth of asphyxia (26–28). Neonates with intrapartum fetal distress had a nearly 5.74 times higher risk of developing birth asphyxia. Similar results have been reported in previous studies in Northwest Ethiopia and Al-Diwaniya (29, 30). A possible reason is that fetal distress is the primary indication of emergency C/S, a known risk factor for birth asphyxia. Assisted vaginal delivery (vacuum or forceps) and C/S delivery pose 2.80 times higher risk of newborn asphyxia compared to mothers with spontaneous vaginal delivery. This result is comparable to the study done in Turkey, China, Nepal, and Cameroon (31–34). The possible reason for this may be newborns delivered by C/S may be either the majority of mothers have had complications, or the decision on C/S may be taken late after complications have occurred, the fetus chest may be stretched when the newborn passes through the birth canal in the vaginal delivery, which may evacuate the secretion. This reduces the chance of developing birth asphyxia (35). Neonates born with low birth weight were 4.20 times more likely to have birth asphyxia than those born with normal birth weight. This finding was supported by previous studies in Iran, Thailand, and Nigeria (36–38). It could be because a high proportion of small babies may be pre-term, after all they may not have adequate surfactants that could contribute to difficulty breathing and having discomfort in the cardiopulmonary transfer and eventually developed birth asphyxia. Concerning fetal presentations, newborns with the non-cephalic presentation were 5.20 times more likely than those with a cephalic presentation to experience birth asphyxia. These findings were supported by the study done in Cameron, Uganda, Nigeria, Thailand (36–39). It may be due to a higher likelihood of umbilical cord prolapse, head entrapment, birth complications, and perinatal mortality (37). Also, neonates born from mothers with co-morbidity during pregnancy were 3.40 times more likely to develop birth asphyxia than their counterpart. This may be because diseases and complications during pregnancy are the most significant risk factors for perinatal mortality, peripartum complications were the most important factors associated with increased risk of birth asphyxia (40, 41).

Newborns born with a gestational age < 37 weeks 2.60 times were more likely to develop birth asphyxia compared to neonates with a gestational age of ≥ 37 weeks. This study is in line with a study conducted in Jordan and Brazil (16, 42). This may be attributed to premature infants who are more vulnerable to ischemia due to incomplete blood-brain barrier development, and preterm infants face multiple morbidities including the organ system, particularly lung immaturity triggering a respiratory failure (43).

In Conclusion this study identified that, the main risk factors of birth asphyxia were fetal distress, instrumental delivery, low birth weight, non-cephalic presentation, preterm, prolonged labor, co-morbidity during pregnancy and meconium tainted amniotic fluid. Most of these factors are preventable through holistic care for pregnancy, labor and delivery. Therefore, to reduce neonatal mortality associated with birth asphyxia, attention should be given to holistic pregnancy care services, labor and delivery care. Furthermore, intervention strategies aimed at reducing birth asphyxia should target the identified factors.

This was the first birth asphyxia study in the study area which shows risk factors of birth asphyxia. The case-control study design that was appropriate to address the research question and enabled the identification of possible risk factors birth asphyxia. This research is quantitative; if qualitative approach was also used it would be stronger to examine the extra risk factors of birth asphyxia in depth. This research may have maternal recall bias as they recalled their previous due to the retrospective nature the design. Using small samples, it's difficult to generalize to society at large. Therefore, larger studies will be needed to demonstrate true associations in the population.
